# Machine Learning Applications in Spine Surgery

**DOI:** 10.7759/cureus.48078

**Published:** 2023-10-31

**Authors:** Themistoklis Tragaris, Ioannis S Benetos, John Vlamis, Spyridon Pneumaticos

**Affiliations:** 1 1st Department of Orthopaedic Surgery, National and Kapodistrian University of Athens School of Medicine, KAT Hospital, Athens, GRC; 2 3rd Department of Orthopaedic Surgery, National and Kapodistrian University of Athens School of Medicine, KAT Hospital, Athens, GRC

**Keywords:** patient reported outcomes, healthcare improvement, machine learning, artificial intelligence, spine, ai & robotics in healthcare

## Abstract

This literature review sought to identify and evaluate the current applications of artificial intelligence (AI)/machine learning (ML) in spine surgery that can effectively guide clinical decision-making and surgical planning. By using specific keywords to maximize search sensitivity, a thorough literature research was conducted in several online databases: Scopus, PubMed, and Google Scholar, and the findings were filtered according to the Preferred Reporting Items for Systematic Reviews and Meta-Analyses (PRISMA) guidelines. A total of 46 studies met the requirements and were included in this review. According to this study, AI/ML models were sufficiently accurate with a mean overall value of 74.9%, and performed best at preoperative patient selection, cost prediction, and length of stay. Performance was also good at predicting functional outcomes and postoperative mortality. Regression analysis was the most frequently utilized application whereas deep learning/artificial neural networks had the highest sensitivity score (81.5%). Despite the relatively brief history of engagement with AI/ML, as evidenced by the fact that 77.5% of studies were published after 2018, the outcomes have been promising. In light of the Big Data era, the increasing prevalence of National Registries, and the wide-ranging applications of AI, such as exemplified by ChatGPT (OpenAI, San Francisco, California), it is highly likely that the field of spine surgery will gradually adopt and integrate AI/ML into its clinical practices. Consequently, it is of great significance for spine surgeons to acquaint themselves with the fundamental principles of AI/ML, as these technologies hold the potential for substantial improvements in overall patient care.

## Introduction and background

The advent of machine learning (ML) applications within clinical medicine signifies the dawn of a new era for addressing healthcare challenges, such as artificial intelligence (AI) tools that can leverage large datasets improving healthcare systems and minimizing human error [1-6]. The field of spine surgery is no exception, where technologies like augmented reality, computer navigation, and robotics are already leaving their mark in both clinical settings and operating rooms [7-9]. Grasping the foundational principles of ML and AI is of paramount importance in effectively and safely unlocking their potential [10-12]. In its early stages, this literature review aims to offer an initial glimpse into the world of ML/AI applications within spine surgery, delving into their objectives, outcomes, and effectiveness [13].

ML, a subset of AI, is dedicated to crafting algorithms that enhance themselves (learners) through experiential learning [14]. Notable instances of AI/ML integration in spine surgery encompass tasks such as image classification (e.g., automating the detection of vertebral compression fractures in CT or MRI scans) [7,15,16], the creation of models for preoperative risk assessment [17-19] and the development of tools to support clinical decision-making [7,10,20].

Boundaries between classic statistics and ML might seem blurry because they are both based on statistical models; however, while the former is derived from mathematics, the latter is derived from computer science. Furthermore, classic statistics infers relationships between variables whereas ML endeavors to predict these [21,22]. Moreover, the inference (in statistics) involves testing the null against an alternative hypothesis for an outcome with a confidence measure, whereas the prediction (in ML) involves predicting outcomes without requiring more prior data because there are derived relationships [23]. As an example, Ogink et al. trained a neural network successfully to predict early and accurately which patients undergoing surgery for spinal stenosis will require admission to a rehabilitation facility after hospital discharge [24].

In spine surgery literature, three of the most commonly used ML applications are: 1. Artificial Neural Networks (ANN), 2. Support Vector Machine (SVM), and 3. Classification and Regression Trees (CART). These applications have some unique and some overlapping features [25,26].

Taking into account the novelty of ML/AI, the aim of this literature review was, on the one hand, to bring the nonexpert reader closer to its terminology and principles and, on the other hand, to elucidate their applications and outcomes regarding spine surgery.

Materials and methods

**Figure 1:**
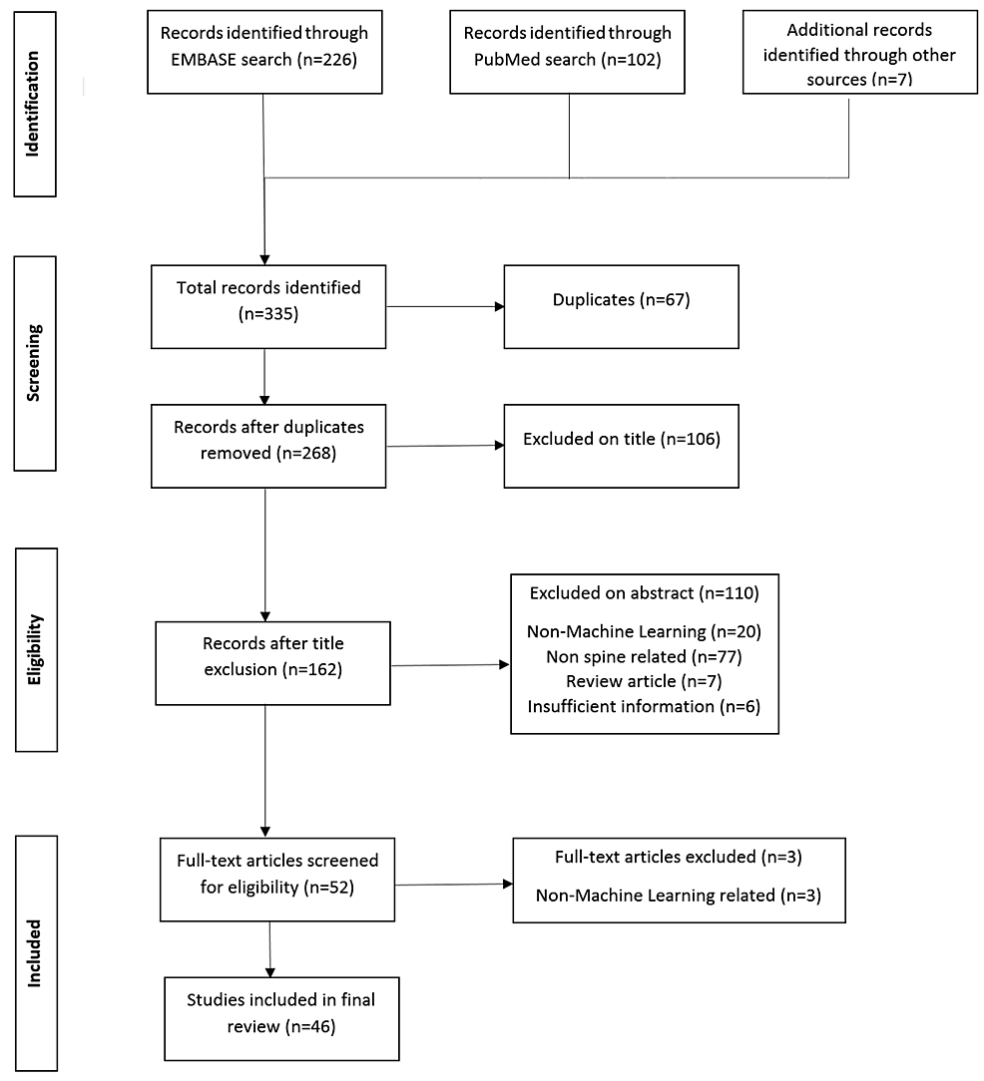
PRISMA study flowchart PRISMA: Preferred Reporting Items for Systematic Reviews and Meta-Analyses

In order to carry out this review, extensive and thorough literature research was conducted by two independent researchers in Scopus, PubMed, and Google Scholar online databases using the following search terms: “machine learning”, “artificial intelligence” “AND” “spine”. Studies’ eligibility for inclusion was assessed based on the Preferred Reporting Items for Systematic Reviews and Meta-Analyses (PRISMA) guidelines (Figure 1). Deviations about inclusion were discussed and settled with consent. No time limit was set for the search.

Original clinical studies investigating and assessing ML/AI applications in spinal surgery were included, while reviews, studies of implant designing and development, non-English language studies, congress lectures, and non-spine surgery-related studies were excluded. Primary databases search resulted in 335 articles and after applying inclusion and exclusion criteria, 46 articles were eligible for final recruitment (Figure 1). Over 80% of the studies were of low level of evidence as per the Oxford Centre for Evidence-Based Medicine (cohort and case-control studies) and 77.5% were published after 2018 (Table 1).

**Table 1:** Studies matching the eligibility criteria of the literature review. ANN=artificial neural networks, BPM=Bayes point machines, BDT=boosted decision tree, CART=classification and regression tree, CNN=convolutional neural network, DNN=deep neural network, GAM=generalized additive model, MARS=multivariate adaptive regression spline, PLS=partial least squares, RF=random forests, SVM=support vector machines, CVE=cardiovascular event, VTE=venus thromboembolism, LOS=length of stay, AUC=area under the curve, ACDF=anterior cervical discectomy and fusion, ASA=American Society of Anaesthesiologists, LR=logistic regression, CHF=congestive heart failure, ODI=Oswestry disability index, ASD= adjacent segment disease

Authors	Models	Cohort	Endpoint	Results
Burns et al. (2017) [7]	SVM	150 CT scans	Vertebral compression fractures detection and localization	98.7% sensitivity with 0,29 false positive
Kalagara et al., 2018 [27]	Lumbar spine/laminectomy	SVM	Readmissions	Accuracy >95%
Karhade et al., 2018 [28]	Deep learning/ANN, decision tree, SVM, Bayesian networks	Lumbar spine degeneration	LOS	Brier score of 0.0713
Khan et al., 2021 [29]	CART, GAM, MARS , PLS , RF, SVM	173 patients with cervical myelopathy	SF-36	AUC 0.74-0.77
Arvind et al., 2018 [30]	Deep Learning/ANN	ACDF	CVE, VTE, 30-day mortality	AUC>0.73
Kim et al., 2018 [31]	Deep Learning/ANN, Regression analysis, SVM	Lumbar decompression	VTE, surgical site infection and 30 day mortality risk	AUC of ANN better than ASA
Kim et al., 2018 [32]	Deep Learning/ANN, Regression analysis, SVM	ASD	VTE, surgical site infection and 30 day mortality risk	ANN and LR outperformed ASA
Durand et al., 2018 [33]	Decision tree	ASD	Blood transfusion	AUC of 0,79
Hopkins et al. (2019) [34]	DNN	23.264 spine fusions	30 day readmission	AUC 0,81
Khatri et al., 2019 [35]	Decision tree	Spinal fusion	Preoperative planning/ selection	Correlation coefficient of 0.96
Kuo et al., 2018 [36]	Deep Learning, SVM, Bayesian point networks	Spine fusion	Cost prediction	AUC=0.904 Accuracy=84.30%
Lerner et al., 2020 [37]	Decision tree	Posterior lumbar interlaminar fusion	2 year costs after fusion	Successful clustering and multivariable correlation
Mehta & Sebro (2020) [38]	SVM	370 DEXA scans	Lumbar spine fracture	SVM detected incidental lumbar spine fractures in DEXA scans with AUC 0,93 and >94% sensitivity and specificity.
Siccoli et al., 2019 [39]	Decision tree	Lumbar spine decompression	LOS and readmissions/reoperations	AUC>0.75
Tee et al., 2019 [40]	CART	806 patients with spinal cord injury	Decision tree analysis	Managed to produce 6 homogeneous groupings
Azimi et al., 2015 [41]	Lumbar disc herniation	Deep Learning/ANN, Regression analysis	Relapse	Accuracy of 95.8%
Azimi et al., 2017 [42]	Lumbar disc herniation	Deep learning/ANN	Favorable outcome postoperatively	Accuracy of 95.8%
Azimi et al., 2014 [43]	Lumbar spine stenosis	Deep Learning/ANN, Regression analysis, SVM	Patient satisfaction	Accuracy of 94.1%
Han et al., 2019 [44]	Regression analysis	Fusion	Adverse events, cardiovascular events/ CHF Surgical complications	AUC of 0,76
Hoffman et al., 2015 [45]	SVM	27 patients with cervical myelopathy	Postoperative ODI score	SVM more accurate than multi linear regression
Hopkins et al., 2020 [46]	DNN	4.046 spine fusions	Surgical site infection	AUC 0,79.
Janssen et al., 2018 [47]	Regression analysis	Thoracolumbar spine procedure	Surgical site infection	AUC of 0.61
Karhade et al., 2019 [48]	Deep Learning/ANN, ανάλυση παλινδρόμησης, SVM, δέντρο αποφάσεων	Spine metastatic disease	90 day and 1 year mortality	c-statistic of 0.89
Karhade et al., 2019 [49]	Deep Learning/ANN, Regression analysis, SVM	ACDF	Opioid use	AUC 0,79.
Karhade et al., 2019 [50]	Spine epidural abscess	Deep Learning/ANN, Regression analysis, SVM	90 day mortality	AUC of 0.75
Karhade et al., 2018 [51]	ANN, BPM, CART, SVM	1.790 cases of metastatic spine disease	30 day mortality	Higher discrimination ability of ANN compared to BPM
Karhade et al., 2019 [52]	penalized logistic regression, random forest, stochastic gradient boosting, ANN, SVM	Spinal Metastatic Disease	90-Day and 1-Year Mortality	Preoperative estimation of 90-d and 1-yr mortality was achieved
Shamim et al., 2009 [53]	Lumbar herniated disc.	Decision tree analysis	Postoperative adverse outcome	Sensitivity>88% Specificity>86%
Karnuta et al., 2019 [54]	Bayesian network	Spinal fusion	LOS cost prediction	AUC>0.880
Ryu et al., 2018 [55]	CART	Spine ependymoma	5 and 10 year mortality	AUC 0,74
Varghese et al., 2018 [56]	CART	27 pedicle screw extraction	Extraction failure	0.99 correlation between observed and predicted outcomes
Vania et al., 2019 [57]	CNN	32 CT scans	Spine segmentation	Sensitivity and specificity >96%.
Liu et al., 2017 [58]	Logistic regression	Cervical spine surgery	rate of cervical spine surgery, cost variation, surgical cost	The rate of cervical spine surgery decreased, mean case cost increased at a rate double that of inflation
Ogink et al., 2019 [59]	Deep Learning, SVM, Bayesian point networks	Spondylolisthesis	LOS	AUC=0.753
Stopa et al., 2019 [60]	ANN	144 patients with lumbar spine procedure	Discharge other than home	AUC 0.89
Bekelis et al., 2014 [61]	Regression analysis	ACDF	VTE, Surgical site infection and 30 day mortality	AUROC > 0.65
Goyal et al.,, 2019 [13]	Fusion	Deep learning/ Decision tree/ SVM	LOS and readmissions/reoperations	AUC>0.8
Staartjes et al., 2019 [62]	Single level microdiscectomy for lumbar spine herniated disc disease	Deep Learning/ANN, Regression analysis, SVM	Regression analysis, SVM	AUC > 0.82
Buchlak et al., 2017 [2]	ASD	Regression analysis	Postoperative complications	AUROC of 0.712
Scheer et al., 2017 [19]	Decision tree	ASD	Adverse events and serious complications	AUROC of 0.89
Khor et al., 2018 [63]	Lumbar spine interlaminar fusion	Regression analysis	Clinical improvement	Concordance statistic 0.66-0.79
Ames et al., 2019 [20]	Decision tree	ASD	Patient type clustering	Successfully constructed a 2-year risk-benefit grid
Huang et al., 2019 [8]	Bayesian networks, SVM, regression analysis	ACDF	Preoperative planning/selection	Accuracy>91.5%
Chia et al., 2017 [10]	Deep learning/ANN	Cerebral palsy	Preoperative planning/selection	Sensitivity>0.70 Specificity>0.80
Assi et al., 2014 [23]	Regression analysis	Scolioisis	Preoperative planning/ reoperations	Mean prediction error of 0.0145
Ogink et al., 2019 [24]	ANN, BDT, BPM , SVM	28.600 patients with lumbar spine procedure	Home discharge	ANN had the highest discrimination ability
Seoud et al., 2010 [16]	SVM	97 juvenile scoliosis	Scoliosis classification (C1, C2 C3)	SVM predicted 72% of cases

## Review

Results

The studies that were finally included in this review were further divided into two main categories: (a) 22 studies regarding the use of ML/AI in assisting clinical decision-making by classification of the given pathology, preoperative patient selection, and preoperative planning (Table 2) [20,27-29] and (b) 24 studies focusing on postoperative outcomes prediction capability of ML/AI (Table 3) [30]. The performance evaluation of the AI/ML model in the examined studies encompassed several measures. These included metrics like the area under the curve (AUC) derived from receiver operating characteristic (ROC) curves, along with accuracy (%), sensitivity (%), and specificity (%) [31,32]. The AUC metric serves as an indicator of the ML model's capacity to distinguish, with its values spanning from 0.50 to 1. A value nearing 1 signifies a heightened predictive ability of the model, while values within the range of 0.51-0.69 suggest less effective performance. Statistical analysis entailed a one-way analysis of variance (ANOVA), followed by subsequent post hoc Tukey tests. The level of statistical significance was predefined as p<0.05.

**Table 2:** ML/AI and preoperative classification, patient selection, and planning in spine surgery ANN=artificial neural networks, BPM=Bayes point machines, BDT=boosted decision tree, CART=classification and regression tree, CNN=convolutional neural network, DNN=deep neural network, GAM=generalized additive model, MARS=multivariate adaptive regression spline, PLS=partial least squares, RF=random forests, SVM=support vector machines, CVE=cardiovascular event, VTE=venus thromboembolism, LOS=length of stay, AUC=are under curve, ACDF=anterior cervical discectomy and fusion, ASA=American Society of Anaesthesiologists, LR=logistic regression, CHF=congestive heart failure, ODI=Oswestry disability index, ASD= adjacent segment disease; ML = machine learning, AI=artificial learning

Author, Date	Pathology/procedure	ML algorithms	Endpoint	Results	Patient number	Database
Burns et al., 2017 [7]	150 CT scans	SVM	Vertebral compression fracture detection and localization	98.7% sensitivity with 0,29 false positive	150	Single center
Kalagara et al., 2018 [27]	Lumbar spine/laminectomy	SVM	Readmissions	Accuracy >95%	4030	ACS-NSQIP
Stopa et al., 2019 [61]	Fusion	Deep Learning/ANN	LOS	AUC of 0.89	144	Single center
Ames et al., 2019 [20]	ASD	Decision tree	Patient type clustering	Successfully constructed a 2-year risk-benefit grid	570	Multicenter ASD
Seoud et al., 2010 [16]	97 juvenile scoliosis	SVM	Scoliosis classification (C1, C2 C3)	SVM predicted 72% of cases	97	Single center
Tee et al., 2019 [40]	806 patients with spinal cord injury	CART	Decision tree analysis	Managed to produce 6 homogeneous groupings	806	Vancouver Rick Hansen Spinal Cord Injury Registry (RHSCIR) between 2004 and 2014
Mehta & Sebro, 2020 [38]	370 DEXA scans	SVM	Lumbar spine fracture	SVM detected incidental lumbar spine fractures in DEXA scans with AUC 0,93 and >94% sensitivity and specificity.	370	Single center dataset
Vania et al., 2019 [58]	32 CT scans	CNN	Spine segmentation	Sensitivity and specificity >96%.	32	several public datasets that were obtained from the Spineweb website and Gangnam Severance Hospital
Goyal et al., 2019 [13]	Fusion	Deep learning/ Decision tree/ SVM	LOS and readmissions/reoperations	AUC>0.8	8872	ACS-NSQIP
Ogink et al., 2019 [60]	Spondylolisthesis	Deep Learning, SVM, Bayesian point networks	LOS	AUC=0.753	1868	ACS-NSQIP
Kuo et al., 2018 [36]	Spine fusion	Deep Learning, SVM, Bayesian point networks	Cost prediction	AUC=0.904 Accuracy=84.30%	532	Single center
Lerner et al., 2020 [37]	Posterior lumbar interlaminar fusion	Decision tree	2 year costs after fusion	Successful clustering and multivariable correlation	18770	IBM MarketScan®
Siccoli et al., 2019 [39]	Lumbar spine decompression	Decision tree	LOS and readmissions/reoperations	AUV>0.75	635	Single center
Chia et al., 2017 [10]	Cerebral palsy	Deep learning/ANN	Preoperative planning/selection	Sensitivity>0.70 Specificity>0.80	242	Single center
Huang et al., 2019 [8]	ACDF	Bayesian networks, SVM, regression analysis	Preoperative planning/selection	Accuracy>91.5%	321	Single center
Varghese et al., 2018 [57]	Spine fusion	CART	Preoperative planning/selection	Correlation coefficient of 0.99	-	Single center
Karhade et al., 2018 [28]	Lumbar spine degeneration	Deep learning/ANN, decision tree, SVM, Bayesian networks	LOS	Brier score of 0.0713	5273	ACS-NSQIP
Hopkins et al., 2019 [34]	Poster ior lumbar interlaminar fusion	Deep learning/ANN	Readmissions/ reoperations	AUC of 0.812	5816	ACS-NSQIP
Ogink et al., 2019 [24]	Lumbar spine stenosis	Deep learning/ANN, decision tree, SVM και Bayesian networks	LOS	AUC of 0.751	9338	ACS-NSQIP
Karnuta et al., 2019 [55]	Spinal fusion	Bayesian network	LOS cost prediction	AUC>0.880	3807	New York State Sparks database
Khatri et al., 2019 [35]	Spinal fusion	Decision tree	Preoperative planning/ selection	Correlation coefficient of 0.96	N/A	Single center
Assi et al., 2014 [23]	Scolioisis	Regression analysis	Preoperative planning/ reoperations	Mean prediction error of 0.0145	141	Single center

**Table 3:** ML/AI and postoperative outcomes prediction models ANN=artificial neural networks, BPM=Bayes point machines, BDT=boosted decision tree, CART=classification and regression tree, CNN=convolutional neural network, DNN=deep neural network, GAM=generalized additive model, MARS=multivariate adaptive regression spline, PLS=partial least squares, RF=random forests, SVM=support vector machines, CVE=cardiovascular event, VTE=venus thromboembolism, LOS=length of stay, AUC=area under the curve, ACDF=anterior cervical discectomy and fusion, ASA=American Society of Anaesthesiologists, LR=logistic regression, CHF=congestive heart failure, ODI=Oswestry disability index, ASD= adjacent segment disease. ML=machine learning, AI=artifical intelligence

Author, Date	Condition/Procedure	ML algorithms	Endpoint	Results	Patient number	Database
Hopkins et al., 2020 [46]	4.046 spine fusions	DNN	Surgical site infection	AUC 0,79.	4046	Single academic center
Karhade et al., 2018 [51]	1.790 cases of metastatic spine disease	ANN, BPM, CART, SVM	30-day mortality	Higher discrimination ability of ANN compared to BPM	1790	
Kim et al., 2018 [31]	Lumbar decompression	Deep Learning/ANN, Regression analysis, SVM	VTE, λοίμωξη τραύματος και 30 ημερών θνησιμότητα	AUC of ANN better than ASA	6789	ACS-NSQIP
Kim et al., 2018 [32]	ASD	Deep Learning/ANN, Regression analysis, SVM	VTE, λοίμωξη τραύματος και 30 ημερών morbidity	ANN and LR outperformed ASA	1746	Βάση ACS-NSQIP
Karhade et al., 2019 [28]	ACDF	Deep Learning/ANN, Regression analysis, SVM	Opioid use	AUC 0,79.	2737	multi-center
Han et al., 2019 [44]	Fusion	Regression analysis	Adverse events, cardiovascular events/ CHF Surgical complications	AUC of 0,76	331870	IBM MarketScan, CMS Medicaid, Medicare
Durand et al., 2018 [33]	ASD	Decision tree	Blood transfusion	AUC of 0,79	205	ACS-NSQIP
Karhade et al., 2019 [52]	Spine metastatic disease	Deep Learning/ANN, ανάλυση παλινδρόμησης, SVM, δέντρο αποφάσεων	90 day and 1 year mortality	c-statistic of 0.89	732	Single center
Scheer et al., 2017 [19]	ASD	Decision tree	Adverse events and serious complications	AUROC of 0.89	557	Multi-center ASD Βάσεις
Janssen et al., 2018 [47]	Thoracolumbar spine procedure	Regression analysis	Surgical site infection	AUC of 0.61	898	NCI SEER
Karhade et al., 2019 [50]	Spine epidural abscess	Deep Learning/ANN, Regression analysis, SVM	90 day mortality	AUC of 0.75	1053	multi-center
Karhade et al., 2019 [28]	Lumbar spine surgery	Regression analysis	Prolonged use of opioids	AUC of 0.76	8435	multi-center
Ryu et al., 2018 [56]	Spine ependymoma	CART	5 and 10 year mortality	AUC 0,74	2822	NCI SEER
Khan et al., 2020 [29]	Degenerative cervical myelopathy (DCM)	Deep Learning/ANN, Regression analysis, SVM	PROMs- (SF-36 MCS, PCS)	AUC >0,72	193	Multicenter trials AOSpine CSM
Staartjes et al., 2019 [62]	Single level microdiscectomy for lumbar spine herniated disc disease	Deep Learning/ANN, Regression analysis, SVM	Regression analysis, SVM	AUC > 0.82	422	Single center
Hoffman et al., 2015 [45]	Cervical myelopathy	Deep Learning/ANN, Regression analysis, SVM	PROMs-ODI	Accuracy > 85%	20	Single center
Shamim et al., 2009 [53]	Lumbar herniated disc.	Decision tree analysis	Postoperative adverse outcome	Sensitivity>88% Specificity>86%	501	Single center
Azimi et al., 2014 [43]	Lumbar spine stenosis	Deep Learning/ANN, Regression analysis, SVM	Patient satisfaction	Accuracy of 94.1%	168	Single center
Azimi et al., 2015 [42]	Lumbar disc herniation	Deep Learning/ANN, Regression analysis	Relapse	Accuracy of 95.8%	402	Single center
Azimi et al., 2017 [41]	Lumbar disc herniation	Deep learning/ANN	Favorable outcome postoperatively	Accuracy of 95.8%	203	Single center
Buchlak et al., 2017 [2]	ASD	Regression analysis	Postoperative complications	AUROC of 0.712	136	Single center
Karhade et al., 2019 [50]	Spinal abscess	Deep Learning/ANN, decision tree, SVM Bayesian Networks	5 year mortality	NN algorithms significantly more accurate than LR	265	NCI SEER
Khor et al., 2018 [63]	Lumbar spine interlaminar fusion	Regression analysis	Clinical improvement	Concordance statistic 0.66-0.79	1965	Multi-center database
Bekelis et al., 2014 [61]	ACDF	Regression analysis	VTE, Surgical site infection and 30-day mortality	AUROC > 0.65	2732	ACS-NSQIP

Regression analysis was the most common ML/AI model applied (58.5%) [54] whereas Bayesian Point Machines had the highest mean AUC score (0.80). AUC was the most frequently utilized accuracy assessment tool (90.2%) and was good for all models overall with a mean score of 0.75 [55-57]. Accuracy was also good overall, mean 74.9% [58]. Deep learning/ANN had the highest mean sensitivity (81.5%) (Table 4). Typically, the ML/AI models demonstrated their strongest performance in tasks related to preoperative patient assessment and planning, as well as cost prediction and estimating the length of hospital stays [59-61]. Additionally, these models exhibited reasonably accurate predictions for postoperative complications, functional outcomes, and clinical results [62-64]. The prediction of postoperative complications and adverse events (cardiovascular, readmissions, reoperations) was less satisfactory with an AUC score of 0.69 and 0.68, respectively (Table 5).

**Table 4:** Statistical comparisons of different ML/AI models ML=machine learning, AI=artificial intelligence

Performance metrics: Mean (SD, N)				
Models AI/ML	AUC	Accuracy	Sensitivity	Specificity
Bayesian Point Networks (BPN)	.80 (.09, 13)	76.9 (11.9, 8)	63.7 (11.0, 4)	67.4 (17.7, 4)
Boosted Ensembled Learning (BEL)	.76 (.10, 13)	74.1 (9.6, 8)	55.7 (21.7, 7)	71.7 (11.4, 7)
Decision Tree (DT)	.77 (.11, 29)	74.0 (8.7, 13)	75.4 (13.7, 12)	62.5 (21.7, 12)
Deep learning/Artificial Neural Network (ANN)	.77 (.11, 34)	83.0 (10.7, 10)	81.5 (12.1, 8)	71.8 (10.1, 8)
Logistic Regression (LR)	.74 (.11, 56)	70.4 (10.6, 13)	70.6 (12.4, 19)	61.0 (12.4, 19)
Support Vector Machines (SVM)	.63 (.18, 17)	67.5 (12.9, 3)	72.3 (18.3, 3)	56.0 (42.9, 3)
ANOVA	P = .007	P = .083	P = .006	P = .554
Tukey post hoc tests	BN vs SVM (P = .009) DT vs SVM (P = .018) ANN vs SVM (P = .019)	– – –	BEL vs ANN (P = .002) – –	– – –

**Table 5:** Statistical results of models about postoperative outcomes/complications prediction

Performance metrics: Mean (SD, N)				
Postoperative predictions/complications	AUC	Accuracy	Sensitivity	Specificity
Cardiovascular events	0.69 (.12, 21)	–	81.0 (4.2, 2)	52.0 (1.4, 2)
Other postoperative complications	0.68 (.12, 31)	85.8 (7.9, 4)	77.6 (4.4, 5)	51.6 (.5, 5)
Postoperative morbidity	0.82 (.08, 30)	–	–	–
Postoperative functional/clinical outcomes	0.75 (.09, 30)	72.2 (11.2, 28)	73.8 (15.5, 24)	60.9 (17.5, 24)
ANOVA	P < .001	P = .027	P = .487	P = .278
Tukey post hoc tests	1 vs 3 (P < .001) 2 vs 3 (P < .001) 3 vs 4 (P = .035)	– – –	– – –	– – –

Discussion

This review comprehensively analyzed and evaluated the prevailing trends in the utilization of ML/AI applications within the realm of spine surgery. The outcomes of these investigations demonstrated an overall positive trajectory, particularly in terms of preoperative planning and cost optimization. This positive trajectory signifies their potential to emerge as a promising tool for ensuring precise and efficient treatment and management for spine patients.

To successfully incorporate ML into the healthcare sector, healthcare practitioners need to acquaint themselves with ML terminology and techniques, such as decision trees, SVM, and ANN. Notably, ML's predictive capabilities shine when dealing with substantial datasets, such as patient-reported outcomes (PROMs)[64]. This was exemplified in Khan et al.'s study [29], where multiple supervised learners accurately predicted improvements in Short Form-36 (SF-36) scores post-surgery for degenerative cervical myelopathy. Their models effectively integrated various factors like comorbidities, examination findings, imaging, and basic characteristics to provide comprehensive predictive insights.

Additionally, a review by Varghese et al. found that ML's potential extends to characterizing the performance of medical devices such as pedicle screws [57]. They used ML to analyze input permutations in their pedicle screw strength protocol. Their study utilized diverse foam densities and angles for pedicle screw insertion, achieving a promising model with low error rates and high predictive accuracy for pedicle screw failure.

Within the scope of this review, 22 studies (47.8%) explored AI/ML applications for classifying pathological findings, optimizing patient selection, and predicting surgical costs related to hospital stays, discharges, readmissions, and other cost factors. Another 24 studies (52.2%) focused on predicting and managing postoperative outcomes, complications, morbidity, mortality, and PROMs, each presenting distinct challenges.

This review stands as a pioneering effort to evaluate and consolidate AI/ML applications for optimizing patient selection, predicting surgical outcomes, and managing complications in spine surgery. Encompassing 46 studies, the review showcases AI/ML-based prediction and optimization models that have the potential to guide clinical decision-making and surgical planning. Across various AI/ML methods, the models demonstrated satisfactory accuracy, averaging 74.9% overall accuracy and an AUC of 0.75. Notably, these models excelled in optimizing preoperative patient selection, planning, cost prediction, hospital discharge, and length of stay. They also performed commendably in predicting postoperative mortality, functional outcomes, and clinical results (AUC between 0.70 and 0.89).

While AI/ML models showed limited success in predicting postoperative complications (AUC 0.50-0.69), they still hold the potential to improve preoperative planning and enhance the cost-effectiveness of healthcare services. Furthermore, the review points out that AI/ML models could help minimize unnecessary healthcare costs and offer models for risk-adjusted reimbursement. It also highlight AI/ML's role in enhancing clinical decision-making precision and patient care, allowing resource optimization for postoperative follow-up and focused care for high-risk patients.

Limitations

The current study is subject to several limitations. Firstly, it is important to acknowledge that the field of ML/AI remains relatively nascent, particularly in its application to spine surgery, and thus its complete impact and potential are yet to be fully realized. Additionally, it is essential to recognize the limited availability of relevant literature, which necessitates a cautious approach when interpreting our findings. Lastly, the retrospective nature of the study introduces inherent limitations that must be duly acknowledged. Notwithstanding the aforementioned limitations, this study contributes to the existing body of literature.

## Conclusions

This review delineates the specific domains within spine surgery where the influence of ML/AI is most pronounced, shedding light on the precise manner in which ML/AI can exert its impact. Furthermore, it serves to bridge the gap between spine surgeons and the emerging field of ML/AI, thereby facilitating a better understanding of its potential applications. Notably, this review provides evidence of promising outcomes stemming from the use of ML/AI in spine surgery, even in its early stages. This observation implies that as the field matures, even more favorable results may be anticipated particularly in supporting and guiding clinical decision-making by powerfully refining the massive data extracted from PROMs and National Registries and improving outcomes overall.

As the field progresses, future research direction should include creating externally validated and commercially viable systems that can seamlessly integrate with existing hospital infrastructures. Additionally, further exploration of optimal methods for identifying surgical candidates from a diverse range of preoperative data is warranted. With the rapid expansion of literature, technology accessibility, and clinical applications, understanding AI/ML-based applications is becoming increasingly crucial in the context of spine surgery. It is important to note that while this review presents statistical findings and trends from recent studies, it does not establish definitive relationships between AI/ML and clinical effectiveness.
